# Winter Quarters

**Published:** 1895-11-16

**Authors:** 


### Winter Quarters.

The coming on of winter is a matter of no small moment to an immense number of people, who, although suffering in the greatest imaginable variety of ways, are tainted with one common malady, and may be roughly described as the tuberculous. Amid some surroundings the human frame is practically impervious to the onslaughts of the microbe of tuberculosis. Among others, it is its willing host, ready at all times to receive it, to suffer from it, and to die in consequence of its ravages; and the really important difference between these two conditions hinges upon the presence or the absence of fresh air. It is not difficult, then, to understand the supreme importance to sufferers from tuberculosis of this oncoming of the winter, for to them it too often means the deprivation of this life-giving necessity, the closing of the windows, the rebreathing of the old foul air, and the steady downward progress which all tuberculous patients know by sad experience attends an English winter. Why do such patients go abroad? Not, surely, for pleasure-seeking and enjoyment? In the highest Alps, in the snuggest spots on the Riviera, roughing it in sailing-boats to the Antipodes, lounging in the soft breezes of Madeira, seeking warmth and dryness in the uplands of South Africa, or amid the Pyramids and desert lands of Egypt—what is it that all these searchers after health discover? what one thing is it that they find amid these infinitely diverse conditions? It is, indeed, one thing, and that of the simplest, viz., the ability to pass a large portion of their time in the fresh air. Few people have any idea of the enormous number of those who are enrolled in the great army of the tuberculous. They are to be numbered by hundreds of thousands, and to all these it is of the utmost moment that the coming on of the winter should not mean being shut up in the house. It is not given to all of us to go to sunny climes. Unfortunately, to very many no loophole of escape is given from their almost certain fate. Die they must. The microbe they cannot avoid, the tendency they cannot eliminate. But there are many to whom the Fates are not so cruel, to whom, if they will but have the energy to secure it, the great panacea, fresh air, is accessible even in this trying English climate. Mr. Treves, speaking of the surgical aspect of tuberculosis, says that the susceptibility or tendency to the disease, the particular factor which renders an individual a tuberculous subject, is not a mere matter of ill-health. It is sometimes hereditary, sometimes a matter of bringing up, sometimes a matter of conditions of life, but in this case largely a matter of fresh air. Fresh air is the great antagonist of the tuberculous tendency, and it is to be borne in mind that this great remedy is not to be looked upon as available only for those who are in comparatively fair health. Mr. Treves says that even in England he has had patients with high temperatures and suppurating wounds, with acute joint mischief and psoas abscesses, out of doors every day in spite of snow, and frost, and rain, and that the good results which he has seen follow the operative treatment of spinal abscess have been due in no small degree to the fact that many of his patients were able to be taken out of doors on a spinal carriage, every day and all day, through the whole of an English winter. "In the open air the tuberculous patient may feel safe." This then is the point which one would impress on that vast number of people who, at the present moment, are thinking chiefly how best they can make themselves comfortable for the winter. Their aim should be rather how to arrange their lives so that, through the dark days of the winter, they may best obtain access to the life-giving fresh air. For those in fair bodily condition walking or riding are available; for the wealthy, driving; for the feebler and the weaker sort, sitting out or even lying in the open. It may not always be pleasant, and the snug warmth of the fireside may draw strongly, as we know it does draw to the destruction of many every year; but the fresh air, even though at the cost of infinite rugs and wraps, is better if health is aimed at.

